# 2022 Herbert Tabor Early Career Investigator Awards: Call for nominations

**DOI:** 10.1016/j.jbc.2022.102516

**Published:** 2022-10-13

**Authors:** Kenneth T. Farabaugh, Alex Toker, George DeMartino

We can taste the science in the air! There have been a ton of great studies published in JBC this past year, and it is now that exciting time of year where we ask you, the scientific community, to help us identify the rising stars in biological chemistry. We want you to get excited about all of the amazing new techniques that were developed, all of the intricate signaling pathways discovered, and all of the extraordinary new theories put forth.

Once again, the 2022 JBC Herbert Tabor Early Career Investigator (JHTECI) Awards, named in honor of long-time editor-in-chief of JBC Herb Tabor, will be given to the first authors of five of the year’s top papers as selected from your nominations by a committee of JBC Associate Editors. The winners will be giving oral presentations at next year’s annual ASBMB meeting, Discover BMB 2023, in Seattle from March 25 to 28, 2023. We are looking forward to an excellent series of talks from these hardworking scientists. You can learn more about the previous winners at the Tabor ECI Awards section on the JBC website.

This is where we need your help—we are asking you to help find the diamonds, the most impactful and high quality science published this year in JBC, and nominate the first authors of those papers for the JHTECI award. When making your nominations, please briefly describe the significance of the work and share any information you may have about the role played by the nominated first author. Additional details can be found here. You can send these nominations to george.demartino@utsouthwestern.edu by October 31st, 2022. Any and all papers published between November 1st, 2021, and October 31st of this year are eligible!

We are grateful for your enthusiasm in recognizing the next generation of young scientists! Thanks for sharing our appreciation of biological chemistry and for continuing to read the JBC.

